# Shotgun Metagenomics of a Water Kefir Fermentation Ecosystem Reveals a Novel *Oenococcus* Species

**DOI:** 10.3389/fmicb.2019.00479

**Published:** 2019-03-13

**Authors:** Marko Verce, Luc De Vuyst, Stefan Weckx

**Affiliations:** Research Group of Industrial Microbiology and Food Biotechnology (IMDO), Faculty of Sciences and Bioengineering Sciences, Vrije Universiteit Brussel, Brussels, Belgium

**Keywords:** shotgun metagenomics, water kefir, microbial diversity, functional potential, *Oenococcus*

## Abstract

Water kefir is a fruity, sour, slightly alcoholic and carbonated beverage, which is made by fermentation of an aqueous sucrose solution in the presence of dried figs and water kefir grains. These polysaccharide grains contain lactic acid bacteria (LAB), yeasts, and sometimes bifidobacteria and/or acetic acid bacteria, which consume sucrose to produce exopolysaccharides, lactic acid, acetic acid, ethanol, and carbon dioxide. Shotgun metagenomic sequencing was used to examine the microbial species diversity present at two time points during water kefir fermentation in detail, both in the water kefir liquor and on the water kefir grains, hence representing four samples. *Lactobacillus harbinensis, Lactobacillus hilgardii, Lactobacillus nagelii, Lactobacillus paracasei*, and a *Lactobacillus* species similar to *Lactobacillus hordei/mali* were present in the water kefir examined, along with *Bifidobacterium aquikefiri* and two yeast species, namely *Saccharomyces cerevisiae* and *Dekkera bruxellensis*. In addition, evidence for a novel *Oenococcus* species related to *Oenococcus oeni* and *Oenococcus kitaharae* was found. Its genome was derived from the metagenome and made available under the name of *Candidatus* Oenococcus aquikefiri. Through functional analysis of the four metagenomic data sets, it was possible to link the production of lactic acid, acetic acid, ethanol, and carbon dioxide to subgroups of the microbial species found. In particular, the production of mannitol from fructose was linked to *L. hilgardii, Candidatus* O. aquikefiri, and *B. aquikefiri*, whereas glycerol production was associated with *S. cerevisiae*. Also, there were indications of cross-feeding, for instance in the case of amino acid supply. Few bacterial species could synthesize a limited number of cofactors, making them reliant on the figs or *S. cerevisiae*. The LAB species in turn were found to be capable of contributing to water kefir grain growth, as dextransucrase-encoding genes were attributed to *L. hilgardii, L. hordei*/*mali*, and *Candidatus* O. aquikefiri.

## Introduction

Water kefir is a fruity, sour, and slightly alcoholic and carbonated, fermented beverage produced by adding water kefir grains to a sucrose solution containing dried figs, and letting the mixture to ferment for 2–4 days at room temperature (De Roos and De Vuyst, 2018). As water kefir fermentations are typically performed at household-level, the conditions vary between fermentations. Most people ferment water kefir at 21–25°C for 24–72 h, using between 6 and 10% sucrose and 6–31% water kefir grains (Gulitz et al., 2011, 2013; Marsh et al., 2013; Laureys and De Vuyst, 2014, 2017; Laureys et al., 2018). The polysaccharide water kefir grains harbor the bacteria and yeasts and part of the water kefir grains is re-used after sieving of the water kefir batch, representing a backslopping process (Laureys and De Vuyst, 2014, 2017). These microorganisms carry out the fermentation of sucrose, thereby producing exopolysaccharides, which are necessary for water kefir grain growth, and lactic acid, acetic acid, ethanol, carbon dioxide, and aroma compounds that give water kefir its flavor and texture.

The water kefir grain microbiota consist of lactic acid bacteria (LAB), mainly *Lactobacillus* species, such as *Lactobacillus casei/paracasei, Lactobacillus hilgardii*, and *Lactobacillus nagelii*, and yeasts, frequently *Saccharomyces cerevisiae*, sometimes bifidobacteria, in particular *Bifidobacterium aquikefiri*, and as a minority also acetic acid bacteria, for instance *Acetobacter fabarum*, the latter especially upon extended fermentation or fermentation in the presence of oxygen (Magalhães et al., 2010; Gulitz et al., 2011, 2013; Marsh et al., 2013; Laureys and De Vuyst, 2014, 2017; Zanirati et al., 2015; Laureys et al., 2016, 2018). In general, water kefir grains are reminiscent of milk kefir grains, which are also consortia of the same major groups of microorganisms in a polysaccharide matrix (De Roos and De Vuyst, 2018). However, milk kefir grains can contain species rarely or not at all found in water kefir, such as *Lactobacillus kefiranofaciens*, which is responsible for the biosynthesis of the heteropolysaccharide kefiran, the main component of milk kefir grains (Prado et al., 2015). In contrast, the main component of water kefir grains is a homopolysaccharide, dextran, produced by *L. hilgardii* (Pidoux, 1989; Waldherr et al., 2010; Laureys and De Vuyst, 2017).

Previous studies on the microbial species diversity of water kefir grains relied on culture-dependent as well as culture-independent techniques, the latter including amplicon sequencing of the V1–V4 and V4–V5 regions of the 16S rRNA gene using metagenomic DNA (Gulitz et al., 2013; Marsh et al., 2013). These metabarcoding studies have indicated the existence of a non-identified *Bifidobacterium* species, which was later identified as *B. aquikefiri* (Gulitz et al., 2013; Laureys et al., 2016), and the predominance of *Zymomonas* species in certain water kefirs (Marsh et al., 2013). However, high-throughput amplicon sequencing often lacks accuracy at the species level, especially when compared to shotgun metagenomic sequencing (Ranjan et al., 2016). Shotgun metagenomic sequencing is witnessing an increased use to unravel the composition of microbial ecosystems involved in food fermentations. So far, it has been employed to investigate the spontaneous fermentation processes of kimchi (Jung et al., 2011), cocoa (Illeghems et al., 2012), puer tea (Lyu et al., 2013; Li et al., 2018), wine (Sternes et al., 2017), sausage (Ferrocino et al., 2018), and beer (Smukowski Heil et al., 2018), as well as of the ecosystem composition of various cheeses (Wolfe et al., 2014; Dugat-Bony et al., 2015; Escobar-Zepeda et al., 2016; Duru et al., 2018), milk kefirs (Nalbantoglu et al., 2014; Walsh et al., 2016, 2018), withered Corvina grapes (Salvetti et al., 2016), an Indian rice wine starter culture (Bora et al., 2016), cereal vinegar (Wu et al., 2017), and a fermented dairy beverage nunu (Walsh et al., 2017). As opposed to amplicon sequencing, shotgun metagenomic sequencing data can further be used to infer potential metabolic functions encoded by the genomes of the members of the ecosystem under study through the assembly of the metagenomic sequence reads followed by gene prediction (De Filippo et al., 2012; Prakash and Taylor, 2012), as has been employed in the cases of cocoa bean fermentation (Illeghems et al., 2015), Mexican ripened cheese (Escobar-Zepeda et al., 2016), cereal vinegar (Wu et al., 2017), sausage fermentation (Ferrocino et al., 2018), and puer tea fermentation (Li et al., 2018). Finally, shotgun metagenomic sequencing analysis of fermented food samples allows the discovery of whole genomes of microorganisms that were not cultured before (Duru et al., 2018; Smukowski Heil et al., 2018).

The aim of this study was to examine in detail the water kefir fermentation ecosystem through shotgun metagenomic sequencing, using both water kefir liquor and water kefir grains at two different time points during the fermentation process to examine whether or not all microbial groups and/or species present were found through culturing before and to enable evaluating changes of these microbial communities over time. The four shotgun metagenomic sequence data sets were used to provide not only a deeper insight into the water kefir ecosystem’s microbial species composition but also to seek its yet undiscovered functional potential, and finally link the potential functions to each member of the ecosystem.

## Materials and Methods

### Water Kefir Fermentation and Sampling

The water kefir fermentation experiment that was initiated by water kefir grains received from a private individual from Lokeren, Belgium, and that was previously assessed as to its microbiology and metabolomics as described before (Laureys and De Vuyst, 2017), was sampled for a metagenomic analysis in the present study. Briefly, a water kefir simulation medium containing unrefined cane sugar (7.1%, m/v) and fig extract (17.6%, v/v) was used for the water kefir fermentations. These were carried out in 100 ml glass bottles containing 85 ml of water kefir simulation medium and 15 g of water kefir grains at 21°C for up to 192 h. At each time point of fermentation, three bottles were removed, the water kefirs were sieved, and the water kefir grains were washed with sterile saline (0.85%, m/v, NaCl; Merck, Darmstadt, Germany) and stored at -20°C. The liquors were centrifuged (7,200 × *g*, 20 min, 4°C) and the cell pellets obtained were also stored at -20°C. Time points 24 and 72 h were selected for metagenomic analysis, based on the outcome of previous studies on these water kefir fermentation processes (Laureys and De Vuyst, 2017).

### Metagenomic DNA Extraction

Metagenomic DNA was extracted from both water kefir liquor pellets and water kefir grains of the samples mentioned above, which resulted in four metagenomes. Pellets from 40 ml of water kefir liquor or 2.2 g of water kefir grains (in 11 aliquots of 0.2 g, due to the high volume of water kefir grains) were used as input material for the DNA extraction protocol that was carried out in tubes. Actual DNA extraction was performed based on a method combining enzymatic, chemical, and mechanical cell lysis, followed by phenol/chloroform/isoamylalcohol extraction and column purification of the DNA, as described previously (Vermote et al., 2018). However, the cell lysis steps also included polysaccharide removal, which was undertaken after the proteinase K treatment and before the chloroform/phenol/isoamylalcohol DNA extraction by adding 100 μl of 5 M NaCl (Merck), incubating the suspension at 65°C for 2 min, adding 80 μl of 10% (m/v) hexadecyltrimethylammonium bromide (Calbiochem, San Diego, CA, United States), and incubating at 65°C for 10 min. Also, in the case of the water kefir grains, the aqueous phases obtained after extraction with chloroform/phenol/isoamyl alcohol from the 11 aliquots were pooled and used for DNA purification.

### Preparation of Libraries, Shotgun Metagenomic Sequencing, and Data Preprocessing

All materials and apparatus were from Thermo Fisher Scientific (Wilmington, DE, United States), unless stated otherwise. After optimizing the shearing time (7, 11, or 15 min), the metagenomic DNA was enzymatically sheared to produce library fragments of the desired length, using an Ion Xpress Plus Fragment Library Kit with 100 ng of DNA as input, and applying the optimal shearing time. The sheared DNA was purified using an Agencourt AMPure XP Kit (Beckman Coulter, Brea, CA, United States), followed by adapter ligation and nick repair, and purification of the adapter-ligated DNA using an Agencourt AMPure XP Kit. The unamplified library was size-selected using an E-Gel SizeSelect 2% agarose gel to produce library fragments of approximately 330 bp. The size-selected library was qualified using a Bioanalyzer 2100 with an Agilent High Sensitivity DNA Kit and quantified using an Ion Library Quantitation Kit based on quantitative PCR. As such, four 200-base-read libraries were prepared for sequencing, further referred to as L24, G24, L72, and G72, where L and G denote the type of input material, i.e., water kefir liquor pellets and water kefir grains, respectively, and the Arab numerals denote the time point, either after 24 or 72 h of water kefir fermentation.

The size-selected library was used as template for emulsion PCR onto Ion Sphere Particles (ISPs), using an Ion PGM Template OT2 400 Kit and the Ion OneTouch 2 Instrument. The template-positive ISPs were enriched using the Ion PGM Enrichment Beads and an Ion OneTouch ES. Template-positive ISPs were loaded on an Ion 316 Chip and sequencing was performed using the Ion Hi-Q Sequencing Kit on an Ion PGM.

The metagenomic sequence data were subjected to quality checks and quality trimming using FastQC v0.10.1 (Andrews, 2010) and PRINSEQ 0.20.2 (Schmieder and Edwards, 2011). All four metagenomic data sets were submitted to the European Nucleotide Archive of the European Bioinformatics Institute (ENA/EBI) and are accessible under the study accession number PRJEB21603.

### Taxonomic Analysis of the Metagenomic Sequence Data

#### Taxonomic Analysis Using All Metagenomic Sequence Reads

The quality-checked metagenomic sequence reads were used to assess the taxonomic composition of the water kefir fermentation samples, using diverse taxonomy profiling tools, namely BLAST (Altschul et al., 1990), Kraken (Wood and Salzberg, 2014), and Kaiju (Menzel et al., 2016).

The BLAST algorithm blastn was used to align the metagenomic sequence reads to sequences in the non-redundant nucleotide (nt) database of the National Center for Biotechnology Information (NCBI, Bethesda, MA, United States) and in a custom-made database consisting of bacterial, archaeal, and fungal genome sequences obtained from RefSeq (NCBI; Tatusova et al., 2014). The BLAST algorithm blastx was used to align the metagenomic sequence reads to sequences in the non-redundant protein (nr) database of the NCBI. The BLAST output was parsed with MEGAN 5.7.0 (Huson et al., 2011), using the following settings: MinScore, 100; MaxExpected, 0.01; TopPercent, 10.0; MinSupport, 150; and LCA percent [i.e., a naive lowest common ancestor (LCA) algorithm], 100. The output from MEGAN was further processed with R (R Core Team, 2017) in RStudio (RStudio Team, 2015), using the packages biom (McMurdie and The Biom-Format Team, 2014), RJSONIO (Temple Lang, 2014), reshape2 (Wickham, 2007), ggplot2 (Wickham, 2009), and RColorBrewer (Neuwirth, 2014).

A custom-made database consisting of archaeal, bacterial, and fungal genome assemblies from type material from the NCBI Assembly database was constructed and used for sequence classification with Kraken. A database consisting of bacterial and microbial eukaryotic protein sequences from nr was used for sequence classification with Kaiju.

#### Taxonomic Analysis Based on DNA Marker Genes

MetaPhlAn2 was used to estimate the composition of the microbial communities during water kefir fermentation based on a comparison of the metagenomic sequence reads to a database consisting of unique clade-specific marker genes (Truong et al., 2015).

In addition, bacterial and archaeal 16S rRNA gene fragments were extracted from the metagenomic data sets with rRNASelector (Lee et al., 2011), and sequence fragments of the internal transcribed spacer sequences ITS1 and ITS2 were extracted with ITSx 1.0.11 (Bengtsson-Palme et al., 2013), using the models for fungi, animals, oomycetes, and higher plants. The former reads were binned using RDP Classifier (Wang et al., 2007), which was trained beforehand with training set No. 14, whereas the latter ones were aligned to the nt database using blastn. The results of both methods were parsed with MEGAN 5.7.0, using the following settings: MinScore, 80; MaxExpected, 0.01; TopPercent, 100; and MinSupport, 10 for the RDP Classifier output; and MinScore, 100; MaxExpected, 0.01; TopPercent, 10; and MinSupport, 10 for the BLAST output.

#### Taxonomic Analysis Based on Metagenomic Recruitment Plotting

To construct metagenomic recruitment plots for species-level taxonomic analysis, genera represented by more than 0.1% of all reads in any of the four metagenomes with any BLAST-based method used were selected. Of all species and subspecies of these genera, the genome sequences of the sequenced type strains were obtained from the NCBI RefSeq assembly database. Another representative strain was chosen, preferably one with a complete genome, if the genome sequence of the type strain of a species was not available. The list of the genome assemblies is represented in Supplementary Table S1. A BLAST search was performed using blastn, using the metagenomic sequence reads as query sequences and the collected genome sequences as database. The minimum identity threshold was set to 60%. Only the top hit for each sequence was retained, using 60% as the minimum query coverage threshold. The result was used as a basis for metagenomic recruitment plotting, using R, RStudio, and the R packages ggplot2, reshape2, and scales (Wickham, 2016).

#### Overall Taxonomic Analysis Based on Tools Using All Metagenomic Sequence Reads and Metagenomic Recruitment Plotting

The results of the bioinformatic analyses using BLAST, Kraken, Kaiju, and metagenomic recruitment plotting were extracted “per read.” Each read was assigned to a taxon by the tools used, in the following order of priority: Kaiju, Kraken, blastn versus nt, blastn versus RefSeq, and blastx versus nr. The genus-level identifications from reads that belonged to the ranks “subspecies,” “species,” and “species group” were extracted from the taxon name and added to the genus-level identifications. Reads from other ranks were summarized in the category “above genus.” Categories represented by less than 0.5% of all reads were summarized in the category “minorities.” Temporal shifts in the microbial communities of the water kefir liquor and the water kefir grains were assessed using the results of the overall taxonomic analysis.

### Metagenomic Sequence Read Assembly, Metagenomic Contig Binning, and Functional Analysis

Assembly of the metagenomic sequence reads into contigs was performed for the four metagenomes separately, as well as for a combined data set comprising all metagenomic sequence reads of the four data sets (co-assembly), using the MEGAHIT assembler (Li et al., 2015), with the preset parameter set “meta sensitive.” Only contigs longer than 1000 bp were retained. Assembly statistics, including total size of the assembly (i.e., the sum of contig lengths), the number of contigs, the longest contig length, the average contig length, the median contig length, and the N50 value were calculated using an in-house Python script. The metagenomic contigs assembled were annotated with Prokka (Seemann, 2014). The Prokka annotations were the basis for the targeted search of relevant metabolic pathways. To facilitate the search for carbohydrate-active enzymes, the predicted products of coding sequences found in the contigs were screened with HMMER 3.1b1^1^ using the dbCAN database (Yin et al., 2012), and using CAZy (Lombard et al., 2014) and CAZypedia, the encyclopedia of carbohydrate-active enzymes ^2^, as references. The features that were found during the examination of the contigs were assigned to corresponding taxa through binning of the metagenomic contigs with CONCOCT (Alneberg et al., 2014) and mapping the contigs or contig fragments produced during binning with BWA-MEM (Li, 2013) to the genome assemblies of species found in the taxonomic analysis. The quality of the resulting bins was assessed with CheckM (Parks et al., 2015).

### Taxonomic Characterization of a Novel *Oenococcus* Species

The taxonomic position of a novel *Oenococcus* species detected through the taxonomic analyses of the metagenomes was further characterized. First, the contigs that were assigned by BWA-MEM to species other than *Oenococcus alcoholitolerans, Oenococcus kitaharae*, and *Oenococcus oeni* but that were clustered into *Oenococcus* metagenomic bins with CONCOCT were discarded, except if they exhibited similarity to any sequence belonging to the genus *Oenococcus* in the NCBI nt database using the blastn algorithm. Then, the contigs carrying rRNA genes related to *Oenococcus* were added to the corresponding bins. These metagenome-assembled genomes (MAGs) were reannotated with Prokka and their assembly statistics were calculated as described above. Based on the assembly statistics, the *Oenococcus* MAG derived from the metagenomic data set L24 was subjected to an assessment with CheckM once more and retained for further analyses. It was uploaded to ENA/EBI and is available under the study accession number PRJEB29525. The protein sequences derived from this MAG, as well as the protein sequences from the genomes of 17 *O. oeni* strains, two *O. kitaharae* strains, one *O. alcoholitolerans* strain, and from *Lactococcus garvieae* ATCC 49156, the latter serving as an outgroup, were used in the analysis with OrthoFinder 1.1.10, making use of DIAMOND as alignment tool (Buchfink et al., 2015; Emms and Kelly, 2015). The resulting set of 326 single-copy core protein sequences was subjected to a multiple sequence alignment using MUSCLE (Edgar, 2004). After trimming, the alignments with trimAl (Capella-Gutierrez et al., 2009) without allowing gaps and concatenating the alignments by using an in-house Python script, a rooted phylogenetic tree was constructed using FastTree (Price et al., 2010). Additionally, the average nucleotide identity (ANI) of the genome assemblies used was calculated with the OrthoANIu tool (Yoon et al., 2017), followed by visualization using FigTree^3^ and the R package ggtree (R Core Team, 2017; Yu et al., 2017).

## Results

### Metagenomic Sequencing

The successful sequencing of the four metagenomic libraries derived from a water kefir fermentation process [two liquor (L) and two grain (G) pellet samples], obtained as a function of time (after 24 and 72 h of fermentation), resulted in four quality-trimmed data sets with a combined size of approximately 1.86 Gbp and with read lengths between 20 and 260 bp (Table 1).

**Table 1:** Statistics of the four metagenomic data sets derived after quality trimming from samples of a water kefir fermentation process as a function of time.

Sample	Metagenome size [Mbp]	Number of reads	Median read length [bp]
L24	438	2,397,600	208
G24	521	2,902,164	198
L72	457	2,239,269	225
G72	445	2,640,708	184
TOTAL	1864	10,194,026	205

*The first letter in the sample code stands for the sample type, namely water kefir liquor (L) or water kefir grains (G). The Arab numbers stand for the sampling time point (sample taken after 24 h or 72 h of fermentation).*

### Taxonomic Analysis of the Water Kefir Metagenomes

#### Taxonomic Analysis Using All Metagenomic Sequence Reads

**FIGURE 1:**
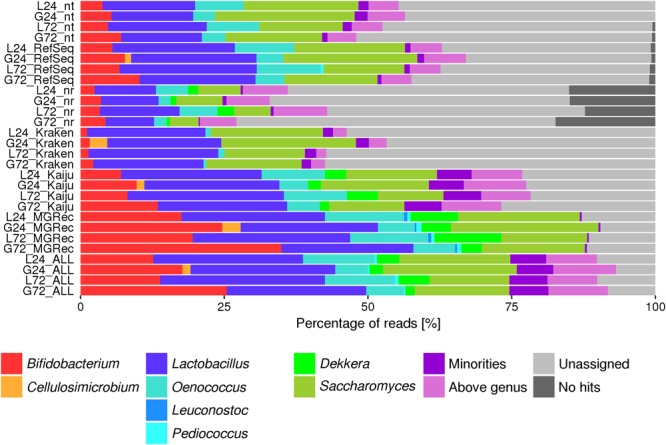
Percentage of metagenomic sequence reads from water kefir fermentation metagenomes assigned to different genera using BLAST [NCBI nucleotide database (nt), RefSeq, or NCBI protein database (nr)], Kraken, Kaiju, metagenomic recruitment plotting (MGRec), or an overall taxonomic analysis (ALL). Actinobacteria are colored in shades of red/orange, Firmicutes in shades of blue, and yeasts in shades of green. The category “minorities” includes all genera that were represented by less than 0.5% of all reads. The category “above genus” represents all assigned taxonomic levels higher than the genus level.

Applying different taxonomy profiling methods to assess the microbial composition of the water kefir fermentation samples resulted in slightly differing taxa and varying percentages of reads assigned to them (Figure 1). The highest number of metagenomic sequence reads were assigned to specific taxa using Kaiju (more than 50% of all reads), whereas the lowest number of reads was assigned using blastx versus nr. Up to 27.2% of all reads were assigned to the genus *Lactobacillus*, and up to 13.5 and 11.0% of all reads to the genera *Bifidobacterium* and *Oenococcus*, respectively. The genus *Pediococcus* was found as a minority in all cases, except for the sample L72 using blastn versus RefSeq (0.55% of all reads). *Cellulosimicrobium* was found in samples G24 and G72, albeit with much fewer reads in the latter (up to 1.3 and 0.01% of all reads, respectively). Among the yeasts, *Saccharomyces* and *Dekkera* were found with all methods, except for Kraken that did not detect *Dekkera*.

#### Taxonomic Analysis Based on DNA Marker Genes

**FIGURE 2:**
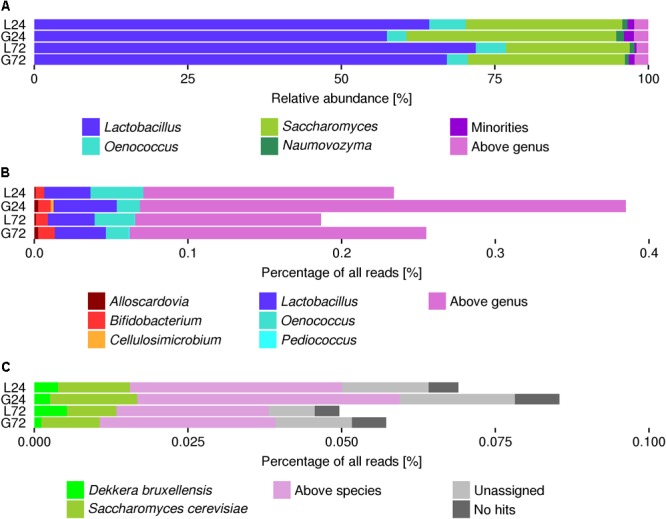
**(A)** Microbial community structure of the water kefir fermentation samples as estimated using MetaPhlAn2, expressed as relative abundances of the microorganisms identified. **(B)** Percentage of metagenomic sequence reads classified into a taxon in the water kefir fermentation metagenomes based on the rRNA gene-related metagenomic sequence reads classified with RDP Classifier. **(C)** Percentage of metagenomic sequence reads classified into a taxon in the water kefir fermentation metagenomes based on the reads from the internal transcribed spacer (ITS) regions classified by means of blastn versus nt. The category “minorities” includes genera with a relative abundance lower than 0.5%. The categories “above genus” and “above species” represent all taxonomic levels assigned higher than the genus level or the species level, respectively.

MetaPhlAn2 estimated that more than half of the microbial communities was represented by species of the genus *Lactobacillus*, followed by *Saccharomyces* (relative abundance of ca. 25%) and *Oenococcus* (relative abundance of less than 10%; Figure 2A). MetaPhlAn2 did not find *Dekkera*, but it predicted the presence of *Naumovozyma* and unclassified *Debaryomycetaceae*.

Reads related to the 16S rRNA gene, which were extracted from the metagenomes with rRNASelector and classified with RDP Classifier (Figure 2B), indicated the presence of *Lactobacillus, Oenococcus*, and *Bifidobacterium* in the four fermentation samples, as well as *Cellulosimicrobium* and *Pediococcus* in some of them. Similarly, the presence of *S. cerevisiae* and *D. bruxellensis* was supported by reads related to ITS1 and ITS2, extracted from the metagenomes with ITSx and aligned to the nt database (Figure 2C).

#### Taxonomic Analysis Based on Metagenomic Recruitment Plotting

To assess the microbial composition of the water kefir ecosystem at the species level through metagenomic recruitment plotting, the metagenomic sequence reads were aligned to a custom-made database consisting of genome sequences of all type strains of those genera that were represented by more than 0.1% of all reads in any of the four metagenomes with any of the three BLAST-based methods applied, namely *Bacillus, Bifidobacterium, Cellulomonas, Cellulosimicrobium, Isoptericola, Lactobacillus, Leuconostoc, Oenococcus, Pediococcus, Sanguibacter, Dekkera*, and *Saccharomyces*.

**FIGURE 3:**
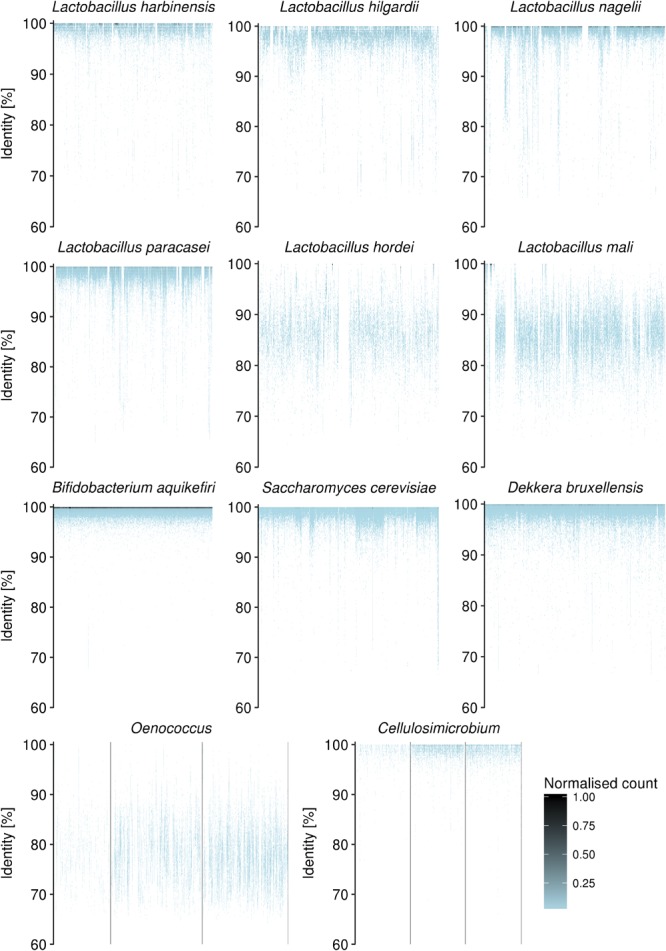
Metagenomic recruitment plots of species or genera found in the water kefir fermentation samples. Each dot represents a bin of metagenomic sequence reads from the data set L24 (water kefir liquor sample after 24 h of fermentation), recruited by a reference genome. The data set L24 is representative for all taxa, except for *Cellulosimicrobium* spp., for which the recruitment of reads from data set G24 (water kefir grains sample after 24 h of fermentation) is shown. The *x*-axis represents the length of a reference genome, or multiple reference genomes in the case of *Oenococcus* spp. (*Oenococcus alcoholitolerans, Oenococcus kitaharae, Oenococcus oeni*) and *Cellulosimicrobium* spp. (*Cellulosimicrobium aquatile, Cellulosimicrobium cellulans*, and *Cellulosimicrobium funkei*). The *y*-axis represents the identity of metagenomic reads to reference sequences. The color represents the number of reads per bin, normalized to the number of reads in the largest bin.

For the data sets L24, G24, L72, and G72, 87.2, 90.4, 88.4, and 88.1% of the metagenomic sequence reads were recruited by the concatenated genome sequences, respectively (Table 2). The *Lactobacillus* genomes recruited approximately 25% of all reads in the four samples. As they recruited reads throughout their lengths and identities close to 100%, *Lactobacillus harbinensis, L. hilgardii, L. nagelii*, and *L. paracasei* were considered to be present (Figure 3). The spread of recruited reads with identities between 80 and 90% throughout the lengths of the *Lactobacillus hordei* and *Lactobacillus mali* genome sequences indicated the presence of at least one species in the samples that was very similar to *L. hordei* and *L. mali* (Figure 3). Accounting for almost all reads recruited by *Bifidobacterium* genomes, the *B. aquikefiri* genome recruited between 17.4 and 34.8% of all reads and was considered to be present (Figure 3). The spread of the recruited reads with identities ranging mainly from 70 to 85% throughout the genome sequences of all three known *Oenococcus* species, namely *O. alcoholitolerans, O. kitaharae*, and *O. oeni*, indicated the presence of a novel *Oenococcus* species that is further referred to as *Candidatus* Oenococcus aquikefiri (Figure 3). The *Cellulosimicrobium* genus was detected in relative abundances higher than 0.5% of all reads in sample G24 solely (Figure 3). Also, 0.02% of all sequence reads from sample G72 were recruited by this genus with high identities. Among the two fungal genera, only *S. cerevisiae* and *D. bruxellensis* were considered to be present, although large parts of the *Saccharomyces pastorianus* genome also recruited reads with up to 100% identity. As their genome sequences did not recruit highly identical reads throughout their lengths, *L. casei, L. kefiranofaciens, Lactobacillus kefiri, Lactobacillus parakefiri*, and species of *Bacillus, Cellulomonas, Isoptericola, Pediococcus*, and *Sanguibacter* were considered not to be present.

**Table 2:** Percentage of all reads of a given metagenomic sequence data set representing water kefir fermentation samples (L, liquor; G, grains) after 24 and 72 h of fermentation, as recruited by the genus mentioned.

Genus	L24	G24	L72	G72
*Bacillus*	0.39	0.26	0.27	0.33
*Bifidobacterium*	17.55	24.66	19.52	34.93
*Cellulomonas*	0.00	0.02	0.00	0.00
*Cellulosimicrobium*	0.00	3.18	0.00	0.02
*Isoptericola*	0.00	0.02	0.00	0.00
*Lactobacillus*	24.98	23.90	27.37	22.93
*Leuconostoc*	0.56	0.31	0.48	0.35
*Oenococcus*	13.73	6.37	13.61	7.24
*Pediococcus*	0.59	0.89	0.59	0.76
*Sanguibacter*	0.00	0.01	0.00	0.00
*Dekkera*	8.28	5.10	11.61	3.60
*Saccharomyces*	21.13	25.71	14.97	17.93
TOTAL	87.20	90.42	88.43	88.09

#### Temporal Shifts of the Microbial Communities in the Water Kefir Liquor and on the Water Kefir Grains

Based on the percentage of metagenomic sequence reads assigned with at least one tool, some shifts in the microbial communities on genus level occurred in the water kefir liquor over time. The percentage of reads assigned to a genus changed most on the water kefir grains, in particular in the case of *Bifidobacterium* with an apparent increase from 17.8 to 25.4%, and in the case of *Saccharomyces* with an apparent decrease from 23.3 to 16.4%. The latter also decreased in the water kefir liquor (19.3–13.9%). The percentage of reads assigned to *Lactobacillus* and *Oenococcus* remained relatively stable. For the former genus, it was around 25% on the water kefir grains and 27% in water kefir liquor, whereas for the latter genus it was around 6% on the water kefir grains and 12% in the water kefir liquor. The percentage of reads assigned to *Dekkera* slightly increased in the water kefir liquor (from 3.9 to 5.4%) and decreased on the water kefir grains (from 2.4 to 1.7%).

### Metagenomic Assembly and Functional Analysis

#### Metagenomic Sequence Read Assembly and Contig Binning

The metagenomic sequence reads of the four data sets, as well as the combined data set, were assembled into contigs to enable *de novo* gene prediction and annotation. The sizes of the assemblies ranged from 24 to 39 Mbp (Table 3). The contigs of the five assemblies were binned into up to 37 bins with CONCOCT. Based on the BWA-MEM mapping of the contigs to reference genomes, almost all bins were associated with at least one of the microbial species, leaving only three bins of up to 27 kbp that could not be associated with any of the species present. Based on CheckM, for most of the bacterial species, in at least one of the assemblies, a bin was found containing contigs that formed a metagenome-assembled genome with more than 50% completeness and less than 10% contamination. Most of the assembled and annotated 16S and 23S rRNA gene sequences were not part of the main bins of the respective bacteria, except in the case of *Candidatus* O. aquikefiri in the G24 assembly. Metagenomic bins assigned to *Oenococcus* were purified and corresponding rRNA gene-containing contigs were added to produce MAGs (Table 4), although no pure *Candidatus* O. aquikefiri bin was obtained from the L72 assembly. The best MAG according to the assembly statistics was that from the L24 assembly, which exhibited a completeness and contamination of 97.86 and 0.18%, respectively.

**Table 3:** Statistics of the assembly of the metagenomic sequence reads into contigs of four water kefir fermentation samples (L, liquor; G, grains) obtained after 24 and 72 h of fermentation.

Sample	Total size of assembly [Mbp]	Number of contigs	Longest contig size [bp]	Average contig size [bp]	Median contig size [bp]	N50 [bp]
L24	29.6	7672	277,860	3863	1720	7332
G24	30.8	6594	252,785	4672	2020	9781
L72	35.6	10,217	215,138	3480	1971	4732
G72	25.4	6119	166,413	4145	2486	5656
ALL	45.1	7326	109,935	6153	3206	10,976

**Table 4:** Assembly statistics of the metagenome-assembled genome (MAG) sequences of the novel *Oenococcus* species, referred to as *Candidatus* Oenococcus aquikefiri.

Source assembly	Total size of assembly [Mbp]	Number of contigs	Longest contig size [kbp]	Average contig size [kbp]	N50 [kbp]	L50 [number of contigs]	CDSs	Average protein length	tRNA genes (amino acids transferable)
ALL	1.52	252	30.3	6.0	8.9	54	1531	283	27 (16)
L24	1.62	75	102.4	21.6	38.9	15	1667	294	43 (20)
G24	1.60	141	57.7	11.4	16.4	30	2016	230	44 (20)
G72	1.62	93	86.8	17.4	25.3	19	1994	239	43 (20)

*The MAG from the metagenome of fermentation sample L24 (water kefir liquor sample after 24 h of fermentation) was deemed the best assembly and used for further analyses.*

#### Carbohydrate Metabolism

Invertase-encoding genes or their fragments were found on contigs assigned to *S. cerevisiae* and *D. bruxellensis*, likely causing the hydrolysis of sucrose into D-glucose and D-fructose during water kefir fermentation, releasing these two monosaccharides for consumption by all capable microorganisms. Genes encoding hexokinases, fructokinases, and the enzymes of the Embden–Meyerhof–Parnas pathway and those for the conversion of pyruvate into ethanol were attributed to both *S. cerevisiae* and *D. bruxellensis*, although some of those genes were not fully assembled in the latter species.

The bacterial members of the water kefir ecosystem of the present study were also able to split sucrose into glucose and fructose, as *L. hordei/mali* possessed three genes, *B. aquikefiri* and *L. harbinensis* two genes, and *L. nagelii, L. paracasei*, and *Candidatus* O. aquikefiri one gene coding for a β-fructofuranosidase/sucrose-6-phosphate hydrolase. Additional fragments of such a gene were associated with *L. harbinensis*. Sucrose phosphorylase-encoding genes were found on contigs attributed to *B. aquikefiri, Candidatus* O. aquikefiri, *L. hilgardii*, and fragments on *L. harbinensis* contigs.

Genes encoding a phosphocarrier protein HPr and an EI component of the phosphoenolpyruvate-dependent phosphotransferase system (PEP-PTS) were associated with *B. aquikefiri* and all LAB members of the water kefir ecosystem of the present study. However, a gene encoding a PTS EIIBCA component that was at least 75% similar to the sucrose-specific PTS EIIBCA components encoded by *Pediococcus pentosaceus* and *Streptococcus mutans* (Swissprot accession numbers P43470 and P12655, respectively) was found only in *L. harbinensis, L. hordei/mali*, and *L. nagelii*, all in the vicinity of a β-fructofuranosidase/sucrose-6-phosphate hydrolase-encoding gene. Genes encoding the mannose-class PTS components EIIAB, EIIC, and EIID, each at least 74% similar to those of *L. casei* (NCBI accession numbers AAY63962, AAY63963, and AAY63964, respectively) or *O. oeni* (NCBI accession numbers ABJ56421, ABJ56422, and ABJ56423, respectively), were attributed to *L. harbinensis, L. hordei/mali, L. nagelii, L. paracasei*, and *Candidatus* O. aquikefiri. Genes encoding proteins similar to a putative glucose uptake protein from *Bifidobacterium animalis* subsp. *lactis* (NCBI accession number ACS48020) were attributed to all LAB members of the water kefir ecosystem of the present study, but not to *B. aquikefiri*.

The heterofermentative pathway to metabolize carbohydrates and produce lactic acid, ethanol, and acetic acid were linked with *L. hilgardii* and *Candidatus* O. aquikefiri, both the hetero- and homofermentative pathways with *L. hordei*/*mali, L. paracasei*, and *L. nagelii*, and the bifidobacterial shunt with *B. aquikefiri.* The core fermentative pathway of *L. harbinensis* could not be fully assembled. Genes encoding mannitol dehydrogenases were found on contigs attributed to *L. hilgardii, Candidatus* O. aquikefiri, and *B. aquikefiri*, making the reduction of fructose to mannitol possible.

Contigs attributed to *S. cerevisiae* contained all necessary genes for glycerol biosynthesis. Genes encoding glycerol kinase and glycerol-3-phosphate dehydrogenase were found on contigs attributed to *Lactobacillus* species, possibly enabling the use of glycerol in their metabolism.

Fragments of genes encoding glucansucrases of glycoside hydrolase family GH70 were attributed to *L. hordei*/*mali, L. hilgardii, L. nagelii*, and *Candidatus* O. aquikefiri. The product of the gene attributed to *L. hilgardii* was 99% identical to the glucansucrase of another *L. hilgardii* strain isolated from water kefir (NCBI accession number CBJ19544.1), although the former lacked the N-terminal repeat-containing region of 218 amino acids found in the latter. The glucansucrase sequences encoded by gene fragments on contigs attributed to the other three species were between 60 and 70% similar to the glucansucrase from *L. hilgardii*.

#### Pyruvate, Citrate, and Malate Metabolism

Genes responsible for the conversion of pyruvate into acetoin were linked with *Lactobacillus* species and *Candidatus* O. aquikefiri, namely through the consecutive action of α-acetolactate synthase and α-acetolactate decarboxylase. Genes encoding diacetyl reductases were linked with *B. aquikefiri, L. hilgardii, L. hordei*/*mali, L. nagelii*, and *Candidatus* O. aquikefiri, enabling the conversion of diacetyl, obtained after spontaneous conversion of α-acetolactate, into acetoin. All *Lactobacillus* species, except for *L. paracasei*, had the genetic means of converting one of the acetoin stereoisomers into a stereoisomer of 2,3-butanediol. Alternatively, pyruvate dehydrogenase genes were linked with *L. hilgardii, L. paracasei, Candidatus* O. aquikefiri, and possibly *L. nagelii*, enabling the decarboxylation of pyruvate into acetyl-CoA. The latter species also possessed a pyruvate synthase gene, potentially enabling it a similar conversion but using oxidized ferredoxin as the electron acceptor. Genes encoding formate *C*-acetyltransferases were associated with *B. aquikefiri, L. hordei*/*mali, L. nagelii, L. paracasei*, and possibly *L. harbinensis*, as the gene was not fully assembled, contributing to formate production by the conversion of pyruvate into acetyl-CoA. Pyruvate oxidase genes were linked with *B. aquikefiri, L. hilgardii, L. paracasei, Candidatus* O. aquikefiri, and possibly *L. harbinensis*, as its gene was not fully assembled, enabling the conversion of pyruvate into acetyl-phosphate and the concomitant production of hydrogen peroxide and carbon dioxide. All LAB and *B. aquikefiri* possessed the acetate kinase and phosphate acetyltransferase genes necessary to convert acetyl-phosphate to either acetate or acetyl-CoA, respectively.

All LAB species present in the water kefir metagenomes possessed genes encoding malic or malolactic enzymes to convert malic acid into lactic acid, whether or not via oxaloacetate and pyruvate. The fumarate hydratase- and fumarate reductase-encoding genes were found in all LAB species, except for *L. harbinensis* and possibly *L. hordei/mali*, due to missing genes or genes not fully assembled, enabling the conversion of malic acid into fumarate, and further into succinate. Genes necessary for citrate lyase activity were attributed to *L. nagelii, L. paracasei*, and *Candidatus* O. aquikefiri, enabling these LAB species to metabolize citrate to oxaloacetate and acetate.

#### Amino Acid Biosynthesis

**FIGURE 4:**
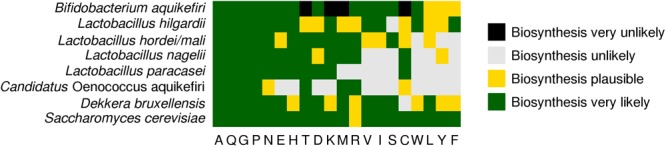
Biosynthetic potential of microbial species found in water kefir to produce amino acids, as assessed through functional analysis of the metagenomes. Black – biosynthesis very unlikely (conclusive evidence of loss-of-function mutations in genes involved in their biosynthesis). Gray – biosynthesis unlikely (at least one gene involved in their biosynthesis not assigned). Yellow – biosynthesis likely (at least one gene involved in their biosynthesis not assembled fully). Green – biosynthesis very likely (all genes involved in their biosynthesis found).

Biosynthetic pathways of between 11 and 19 amino acids were reconstructed for all members of the water kefir ecosystem of the present study, except for *Candidatus* O. aquikefiri and *L. harbinensis*, whose pathways were reconstructed for only seven and two amino acids, respectively (Figure 4). Therefore, *L. harbinensis* was excluded from the following amino acid biosynthesis assessment. Complete biosynthetic pathways for all amino acids, except for L-arginine, were found for *S. cerevisiae*. All other members of the water kefir ecosystem of the present study possessed the enzyme-encoding genes to produce L-alanine from either L-cysteine (all microbial species) or pyruvate (*L. paracasei* and *S. cerevisiae*), L-glutamine from L-glutamate, L-proline from L-glutamate, and L-glycine from L-serine. The biosynthetic pathways for L-tyrosine and L-leucine were not reconstructed fully for any of the LAB species. Genes for chorismate biosynthesis were found in *L. hilgardii* and *L. nagelii*, but a transaldolase gene was not found in any of the LAB species. The transaldolase gene is necessary to produce the precursor erythrose 4-phosphate through the pentose phosphate pathway. In contrast, genes for the full biosynthetic pathways of aromatic amino acids were found for *S. cerevisiae* and *B. aquikefiri*, although the 3-phosphoshikimate 1-carboxyvinyltransferase gene of the latter was not found in full length. A nonsense mutation in the aspartate kinase gene of *B. aquikefiri* made it incapable of L-threonine, L-methionine, and L-lysine biosynthesis.

#### Vitamin and Cofactor Biosynthesis

Full biosynthetic pathways for phosphopantothenate, tetrahydrofolate, thiamine pyrophosphate, and riboflavin by *S. cerevisiae* were found, whereas the biosynthetic pathways for NAD(P)H and pyridoxal 5-phosphate contained some incomplete genes. Due to presumably incomplete or absent biosynthetic pathways, as observed by the absence of several genes per pathway, the bacterial species would depend on the precursors pantothenate, thiamine, and folic acid to produce phosphopantothenate, thiamine pyrophosphate, or tetrahydrofolate, respectively, with the possible exception for tetrahydrofolate in the case of *L. hilgardii*. The capability of pyridoxal 5-phosphate production from glutamate and glyceraldehyde 3-phosphate was found for *B. aquikefiri, L. hilgardii, L. paracasei*, and *Candidatus* O. aquikefiri. The capability of NADH biosynthesis from aspartate was found for *B. aquikefiri* and *L. hilgardii*, whereas the other bacterial species would need nicotinate or β-nicotinate D-ribonucleotide as precursors.

### Taxonomic Characterization of the Novel *Oenococcus* Species

**FIGURE 5:**
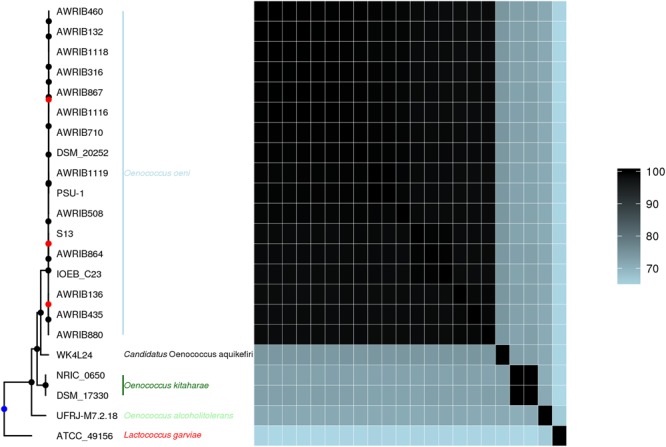
A rooted phylogenetic tree based on single-copy core amino acid sequences from 21 strains of different *Oenococcus* species and *Lactococcus garvieae* as outgroup, accompanied by average nucleotide identities between the genomes of the same strains. The blue dot on the phylogenetic tree denotes the rooting point, the black dots denote nodes with a support value higher than 0.95, and the red dots denote nodes with a support value lower than 0.95.

The core genome of the *Oenococcus* strains in the present analysis consisted of 615 orthogroups containing between 633 (*O. oeni* IOEB_C23) and 680 (*O. alcoholitolerans* UFRJ-M7.2.18) coding sequences. Among the 142 orthogroups present solely in *Candidatus* O. aquikefiri, 128 encoded hypothetical proteins, whereas the rest included genes encoding an arsenate reductase, a 2-hydroxy-3-oxopropionate reductase, a 5,10-methylenetetrahydrofolate reductase, a 5-methyltetrahydropteroyltriglutamate–homocysteine methyltransferase, an α-xylosidase, a putative hydrolase, an ATP-dependent Clp protease ATP-binding subunit ClpE, a ribonuclease R, a putative glycosyltransferase, a putative ribosomal *N*-acetyltransferase, an amidophosphoribosyltransferase, a uric acid permease, a quaternary ammonium compound-resistance protein, a peptide chain release factor 3, and a sensor histidine kinase. Conversely, 17 orthogroups were represented in all *Oenococcus* genomes analyzed, except for *Candidatus* O. aquikefiri. A phylogenetic tree constructed from single-copy core amino acid sequences common to the three known *Oenococcus* species, *Candidatus* O. aquikefiri, and *L. garvieae* as an outgroup, showed that the *Oenococcus* species found in the water kefir branched between *O. oeni* and *O. kitaharae*, with the ANI with strains of those species between 70 and 75% (Figure 5). The assembled 16S rRNA gene sequences belonging to *Candidatus* O. aquikefiri were 97% identical to the *O. oeni* JCM 6125 and *O. kitaharae* NRIC 0645 16S rRNA gene sequences (NCBI accession numbers LC071842.1 and NR_041312.1, respectively).

## Discussion

The taxonomic analysis of four water kefir metagenomes derived from two time points during a water kefir fermentation process, both water kefir liquors and water kefir grains, revealed a rather restricted bacterial diversity and an even more restricted yeast diversity. This is in agreement with culture-dependent data reported before (Magalhães et al., 2010; Gulitz et al., 2011; Laureys and De Vuyst, 2014, 2017) and even for the same water kefir fermentation processes (water kefir C in Laureys and De Vuyst, 2017). The metagenomic analysis of both water kefir liquors and water kefir grains of the present study revealed *Lactobacillus, Oenococcus*, and *Bifidobacterium* as the major bacterial genera, and *Saccharomyces* and *Dekkera* as the major yeast genera.

Using combinations of several tools and databases to obtain an overview of genus-level microbial diversity seemed to be a good approach to assess the composition of a microbial ecosystem. For instance, relying solely on Kraken or MetaPhlAn2 would result in an underestimation or even complete exclusion of *Dekkera*. Indeed, only sequences of *Dekkera custersiana* (*Brettanomyces custersianus*) were included in the Kraken database and no *Dekkera* marker genes were present in the MetaPhlAn2 database, since only a limited number of *Dekkera* sequences are available in public databases. Whereas in general shotgun metagenomics allows obtaining a good insight into the taxonomic composition of an ecosystem at genus level, species-level results heavily depend on the taxonomic analysis tools used (Illeghems et al., 2012). To circumvent this, metagenomic recruitment plots can be used based on the genome sequences of type strains, if possible, as type strains are the basis of species descriptions and nomenclature according to the Bacteriological Code (1990 Revision; Lapage et al., 1992). Indeed, based solely on the alignment of metagenomic sequence reads to sequences in public databases, species not present in the samples could be mistakenly declared as present, due to read alignment to conserved genes or genomic segments horizontally transferred from another species (Gogarten et al., 2002). Based on all taxonomic analysis tools used, including metagenomic recruitment plots, the bacterial species *B. aquikefiri, Candidatus* O. aquikefiri, *L. harbinensis, L. hilgardii*, a possibly novel *Lactobacillus* species related to *L. hordei* and *L. mali, L. paracasei*, and the yeast species *D. bruxellensis* and *S. cerevisiae* were considered to be present in all four water kefir metagenomes for both water kefir liquors and water kefir grains. These results are in agreement with previous studies that have reported the presence of LAB, most commonly lactobacilli of the *L. casei* group (e.g., *L. paracasei*), the *Lactobacillus salivarius* group (e.g., *L. nagelii, L. hordei*, and *L. mali*), and the *Lactobacillus buchneri* group (e.g., *L. hilgardii*), and yeasts, most commonly *S. cerevisiae*, whereas other LAB species, bifidobacteria (e.g., *B. aquikefiri*), acetic acid bacteria (e.g., *A. fabarum*), and *D. bruxellensis* occur less frequently (Moinas et al., 1980; Pidoux, 1989; Galli et al., 1995; Franzetti et al., 1998; Magalhães et al., 2010; Waldherr et al., 2010; Gulitz et al., 2011, 2013; Miguel et al., 2011; Hsieh et al., 2012; Marsh et al., 2013; Laureys and De Vuyst, 2014, 2017; Zanirati et al., 2015; Laureys et al., 2016, 2018). Indeed, all *Lactobacillus* species found in the present study have also been found culture-dependently (*L. paracasei, L. hilgardii*, and *L. harbinensis*) or culture-independently (all of them) in the same water kefir (C) examined previously (Laureys and De Vuyst, 2017). Also, *Cellulosimicrobium cellulans* was detected in the metagenomes derived from water kefir grains but not in those from water kefir liquor.

Species of the *L. casei* group commonly occur in water kefir (Pidoux, 1989; Galli et al., 1995; Magalhães et al., 2010; Gulitz et al., 2011, 2013; Miguel et al., 2011; Zanirati et al., 2015), although nomenclature of these species is problematic due to difficulties in distinguishing them phenotypically and even by 16S rRNA gene sequencing (Wuyts et al., 2017). As such, most strains designated as *L. casei* or *L. paracasei* are members of one species that is currently called *L. paracasei* (Judicial Commission of the International Committee on Systematics of Bacteria, 2008; Smokvina et al., 2013; Wuyts et al., 2017). The water kefir ecosystem of the present study contained *L. paracasei*, not *L. casei*, as shown through metagenomic recruitment plotting. *L. hilgardii*, a species frequently reported in water kefirs, and *L. nagelii* were first isolated from wines (Douglas and Cruess, 1936; Pidoux, 1989; Edwards et al., 2000; Waldherr et al., 2010; Gulitz et al., 2011, 2013; Laureys and De Vuyst, 2014, 2017; Zanirati et al., 2015). A *Lactobacillus* species closely related to *L. hordei* and *L. mali*, currently without a known genome sequence, was present in the water kefir samples of the present study. As *L. mali* and *L. hordei* are commonly found in water kefirs (Hsieh et al., 2012; Gulitz et al., 2013; Laureys and De Vuyst, 2014, 2017; Zanirati et al., 2015) and were first isolated from cider and malted barley, respectively (Carr and Davies, 1970; Rouse et al., 2008), it is not surprising that a similar species would be found in water kefir. So far, *L. harbinensis*, a species that was first isolated from traditional fermented vegetables, has only been reported in Belgian water kefirs (Miyamoto et al., 2005; Laureys and De Vuyst, 2014, 2017).

*Bifidobacterium aquikefiri* is a recently described bifidobacterial species isolated from a water kefir having a common ancestor with the water kefir studied here (Laureys et al., 2016). According to the 16S rRNA gene sequences available in public databases, *B. aquikefiri* was previously detected culture-independently through amplicon sequencing of water kefir grains (Gulitz et al., 2013). Also, *B. psychraerophilum*, a relative of *B. aquikefiri*, has previously been associated with water kefir (Hsieh et al., 2012; Gulitz et al., 2013; Laureys and De Vuyst, 2014). *Bifidobacteriaceae* have also been previously detected in yet another water kefir, albeit on family level only (Marsh et al., 2013).

The yeast species *S. cerevisiae* and *D. bruxellensis* are frequently reported in water kefir, the former being widespread and the latter occurring less frequently (Moinas et al., 1980; Galli et al., 1995; Franzetti et al., 1998; Magalhães et al., 2010; Gulitz et al., 2011; Miguel et al., 2011; Hsieh et al., 2012; Marsh et al., 2013; Laureys and De Vuyst, 2014, 2017). The assignment of metagenomic sequence reads to *S. pastorianus* was not surprising, as this yeast species is an interspecies hybrid between *S. cerevisiae* and *Saccharomyces eubayanus* (Libkind et al., 2011). Both yeast species found in water kefir appear also in other fermented (alcoholic) beverages, such as wines and ales, whereby *S. cerevisiae* is the main fermentative microorganism. In wine, *D. bruxellensis* is considered as a spoilage microorganism, although it is highly functional and desired in lambic beer (De Roos and De Vuyst, 2018; De Roos et al., 2018).

Although long-term stability of water kefir is assumed, the most prominent temporal changes were the decrease in relative abundance of *S. cerevisiae* and an increase in relative abundance of *B. aquikefiri*. This might be due to a fast growth of *S. cerevisiae* in a carbohydrate-rich environment, which are the conditions at the beginning of water kefir fermentations. It might also be that *B. aquikefiri* prefers growth conditions that are only met later in the water kefir fermentation, for example the presence of metabolic by-products of other microorganisms in the ecosystem. Alternatively, *B. aquikefiri* might grow better than other microorganisms in this water kefir ecosystem under certain limiting conditions. For instance, the potential of *B. aquikefiri* to produce tryptophan could give it an advantage upon tryptophan depletion, as tryptophan is the least abundant amino acid in figs (USDA, 2018).

The presence of *Cellulosimicrobium* species on the water kefir grains has not been reported in water kefir before, although ingredients such as figs contain cellulose. However, the enzyme preparations lyticase and Zymolyase used in the DNA extraction protocol are derived from cultures of *Arthrobacter luteus*. That name, along with *Cellulomonas cellulans* and *Oerskovia xanthineolytica*, is one of the older names of *C. cellulans* (Schumann and Stackebrandt, 2012). Hence, *Cellulosimicrobium* can be traced back to the materials used for DNA extraction. Indeed, DNA extraction kits and other laboratory reagents have previously been reported as a source of contaminating DNA (Salter et al., 2014). Moreover, due to the pooling of multiple aliquots during the DNA extraction in the case of the water kefir grains, the contribution of contaminating DNA from the enzymes was higher in the water kefir grain samples than in the water kefir liquor samples.

Apart from the water kefir grains, which serve as the inoculum, the major substrate inputs into the water kefir ecosystem were sucrose and dried figs. The sucrose added, as well as the glucose and fructose from the dried figs, were the main carbon and energy sources for microbial growth. Assuming the capability of transport, all microbial species present were predicted to metabolize sucrose, resulting in the production of lactic acid, acetic acid, ethanol, carbon dioxide, and exopolysaccharides, whose production has been shown experimentally in the water kefir under study previously (Laureys and De Vuyst, 2017). Mannitol production by reduction of fructose, frequently carried out by heterofermentative LAB species (Gänzle, 2015) and linked to *L. hilgardii, Candidatus* O. aquikefiri, and *B. aquikefiri*, and glycerol production associated with *S. cerevisiae* have also been shown experimentally in the water kefir under study previously (Laureys and De Vuyst, 2017). Based on the presence of a specific tryptophan residue, the glycoside hydrolase family GH70 proteins were likely classical sucrases as opposed to glucanotransferases (Brison et al., 2012; Gangoiti et al., 2017). Thus, a dextran could likely be produced from sucrose by *L. hilgardii*, which is known to be a species involved in water kefir grain formation (Brison et al., 2012; Gangoiti et al., 2017), and also by *L. hordei*/*mali, L. nagelii*, and *Candidatus* O. aquikefiri. Whether or not the latter three or other LAB species make major contributions to water kefir grain formation and growth is not known so far. However, recent studies on the structure and function of polysaccharides in water kefir grains and liquor have shown that dextrans with varying branching are present and are likely produced by different LAB species, whereby a dextran produced by *L. hordei* induces aggregation of *S. cerevisiae*, possibly making them more likely to attach to water kefir grains *in vivo* (Fels et al., 2018; Xu et al., 2018).

The figs were the main source of macro- and micronutrients, including nitrogen (amino acids from the protein fraction), sulfur, phosphorus, vitamins, and minerals, besides sucrose. Differences in the amino acid biosynthesis capabilities between microbial species may make them depend on each other to supply limiting amino acids, such as tryptophan, tyrosine, methionine, and histidine, by the conversion from other amino acids that are more plentiful in the figs, such as glutamate and aspartate (USDA, 2018). For example, with the possible exception of *L. hilgardii* in the case of all three aromatic amino acids and *L. nagelii* in the case of L-tyrosine, none of the LAB species reported were predicted to synthesize aromatic amino acids, which could be supplied by the dried figs or, when those were depleted, by *S. cerevisiae* or possibly *B. aquikefiri*. Similarly, LAB species likely depended on the figs or *S. cerevisiae* for their vitamin and cofactor requirements. Indeed, it has previously been reported that *L. hordei* and *L. nagelii* benefit from amino acids and pyridoxal phosphate released by the yeast *Zygotorulaspora florentina* (Stadie et al., 2013).

Given the high completeness and low contamination, the metagenome-assembled genome of *Candidatus* O. aquikefiri obtained in the present study would fulfill the criteria for a high-quality draft according to the genome reporting standard MIMAG, if all rRNA genes were present (Bowers et al., 2017). However, the presence of *Lactobacillaceae* interfered with the assembly or binning of rRNA genes. The phylogenetic tree, the ANI values, and the assembled 16S rRNA gene sequences all supported the novelty of the species found. To our knowledge, there is only one other report of an *Oenococcus* species in water kefir, namely *O*. *oeni* and *O. kitaharae*, which were identified based on 16S rRNA gene sequencing and amplified ribosomal DNA restriction analysis, respectively (Zanirati et al., 2015). Whereas *O. oeni* is typically found in wine (Sternes and Borneman, 2016), *O. kitaharae* and *O. alcoholitolerans* were isolated from a composting distilled shochu residue, and cachaça and ethanol fermentation processes, respectively (Endo and Okada, 2006; Badotti et al., 2014). The *Oenococcus* species found in the metagenomes of the present study was different from all three species mentioned above. As no *Oenococcus* isolates were retrieved in culture-dependent studies of this water kefir previously (Laureys and De Vuyst, 2017), the further description of the *Candidatus* O. aquikefiri species still needs to be performed after successful isolation.

## Conclusion

The water kefir ecosystem of the present study contained *B. aquikefiri, L. harbinensis, L. hilgardii, L. nagelii, L. paracasei, D. bruxellensis*, and *S. cerevisiae*. Notable temporal shifts occurred only in relation to *S. cerevisiae* and *B. aquikefiri*, indicating their preference for growth earlier and later in the fermentation, respectively. This study further showed that a possibly novel *Lactobacillus* species related to *L. hordei* and *L. mali* as well as a novel *Oenococcus* sp., *Candidatus* O. aquikefiri, were present. Whereas the sequencing data were not conclusive for the novelty of the former LAB species, the novelty of the latter was well supported by the metagenomic sequence analyses. The functional analysis of the metagenomic data linked the production of metabolites during water kefir fermentation to certain species or group(s) of species. It showed that the microbial species present in the water kefir ecosystem studied had different capabilities of amino acid, vitamin, and cofactor biosynthesis, indicating possible cross-feeding interactions and the necessity of using dried figs (or other fruits) as a provider of macro- and micronutrients.

## Data Availability

The datasets generated for this study can be found in European Nucleotide Archive, PRJEB21603 (metagenomic data sets) and PRJEB29525 (metagenome-assembled genome).

## Author Contributions

MV performed the metagenomic sequencing and subsequent bioinformatics analyses. SW and LDV designed and coordinated the study. MV, SW, and LDV wrote the manuscript. All authors read and approved the final manuscript.

## Conflict of Interest Statement

The authors declare that the research was conducted in the absence of any commercial or financial relationships that could be construed as a potential conflict of interest.

## Footnotes

1 http://hmmer.org/

2 www.cazypedia.org

3 http://tree.bio.ed.ac.uk/software/figtree/
